# An intelligent healthcare framework for hepatocellular carcinoma diagnosis based on aggregated learners from biomedical data utilising explainable artificial intelligence

**DOI:** 10.1038/s41598-026-39871-z

**Published:** 2026-02-16

**Authors:** Bassam A. Y. Alqaralleh, Malek Zakarya Alksasbeh, Atik Kulakli, Aymen I. Zreikat

**Affiliations:** 1 College of Business Administration, American University of The Middle East, Egaila, Kuwait; 2 Computer Information Systems Department, College of Information Technology, Al Hussein Bin Talal University, Ma’an, Jordan; 3 College of Engineering and Technology, American University of the Middle East, Egaila, Kuwait

**Keywords:** Hepatocellular carcinoma, Ensemble deep learning, Biomedical data processing, Explainable artificial intelligence, Smart healthcare, Cancer, Computational biology and bioinformatics, Mathematics and computing

## Abstract

In recent days, biomedical data mining and machine learning (ML) technologies have transformed the healthcare sector, which utilises cutting-edge medical innovative tools to develop effective decision support systems for disease diagnosis and health informatics. Liver cancer (LC) is a major contributor to the global cancer problem. Incidence rates of this disease have improved in several countries in the past decades. As the main histological kind of LC, hepatocellular carcinoma (HCC) constitutes the large majority of LC diagnoses and deaths. HCC is one of the primary reasons for cancer occurrence and fatality. Initial diagnosis of HCC remains the main aim in improving the poor diagnosis of this type of LC. Recognising HCC at an initial stage is frequently related to improved treatment possibilities for patients with small and symptomless tumours. Several artificial intelligence (AI) methods are considered advanced methods for processing and handling composite multimodal data ranging from repetitive clinical variables to higher-resolution medical images. This paper presents a Hepatocellular Carcinoma Diagnosis based on an Aggregated Learners Utilising Explainable Artificial Intelligence (HCDAL-XAI) model from biomedical data. The primary purpose of the HCDAL-XAI model is to deliver an accurate detection model for initial diagnosis and efficient treatment of HCC using progressive methods. Initially, the data pre-processing step uses min-max normalisation. Furthermore, the HCDAL-XAI model employs an ensemble of a sparse autoencoder (SAE), gated recurrent unit (GRU), and deep belief network (DBN) for the classification process. Lastly, the explainable AI (XAI) model employs SHapley Additive exPlanations (SHAP) to enhance the reliability of AI methods by making their decision-making processes understandable to humans. The comparison analysis of the HCDAL-XAI methodology portrayed a greater accuracy value of 98.18% over existing models under the HCC dataset.

## Introduction

As per the information provided by the World Health Organisation (WHO), about 14.1 million people are identified as having cancer annually, which leads to 8.2 million fatalities worldwide. An LC known as HCC develops from cirrhosis and chronic liver disorder^1^. Current researches signify that HCC is among the deadliest cancers globally, causing around 600,000 deaths every year. Moreover, LC ranks in the 6th position in the most commonly diagnosed tumours globally^2^. Therefore, it is vital to decrease the death rates caused by HCC, which can only be attained by earlier identification. Over the last ten years, growth in population sciences, cellular biology, and molecular and genetic science has led to significant progress in understanding molecular pathways and epidemiologic risk factors that cause liver carcinogenesis^3^. Progress in imaging, treatment, interventional radiology, medical device development, liver transplantation, and surgical techniques has also led to considerable advances in local ablation methods, surgical therapy, and locoregional therapy for HCC. Those advancements have led to significant possibilities to prevent, monitor, diagnose, predict, and treat HCC^4^.

Chronic liver disease and cirrhosis are the most critical risk factors for HCC growth, with viral hepatitis and extreme alcohol consumption being the primary risk factors globally. The occurrence of HCC has a broad geographical difference because of the vast heterogeneity of the risk factor penetration in the population^5^. Any individual who has chronic liver injury and cirrhosis is at risk of cancer development. The most relevant are hepatitis C (HCV) as well as hepatitis B (HBV) viruses, and alcohol. Other reasons for hepatocyte damage are copper or iron deposition, primary biliary cholangitis, and non-alcoholic steatohepatitis, which are also risk factors, but their minor occurrence reduces their entire significance. HBV infection is widespread in underdeveloped countries^6^. HCV is a blood-transmitted agent, which is the primary risk factor. It joins with alcohol consumption. The increasing frequency of HCC in developed nations is a result of the 20 to 40-year age range. Ultrasound (US) analysis plays a significant role in recognising HCC and can identify tiny lesions in the liver^7^. Current practice has concentrated on using Magnetic Resonance Imaging (MRI) and Computed Tomography (CT) with multiphase contrast enhancement. HCC is typically identified by its different echogenicity from the surrounding liver on US analysis^8^.

**Fig. 1:**
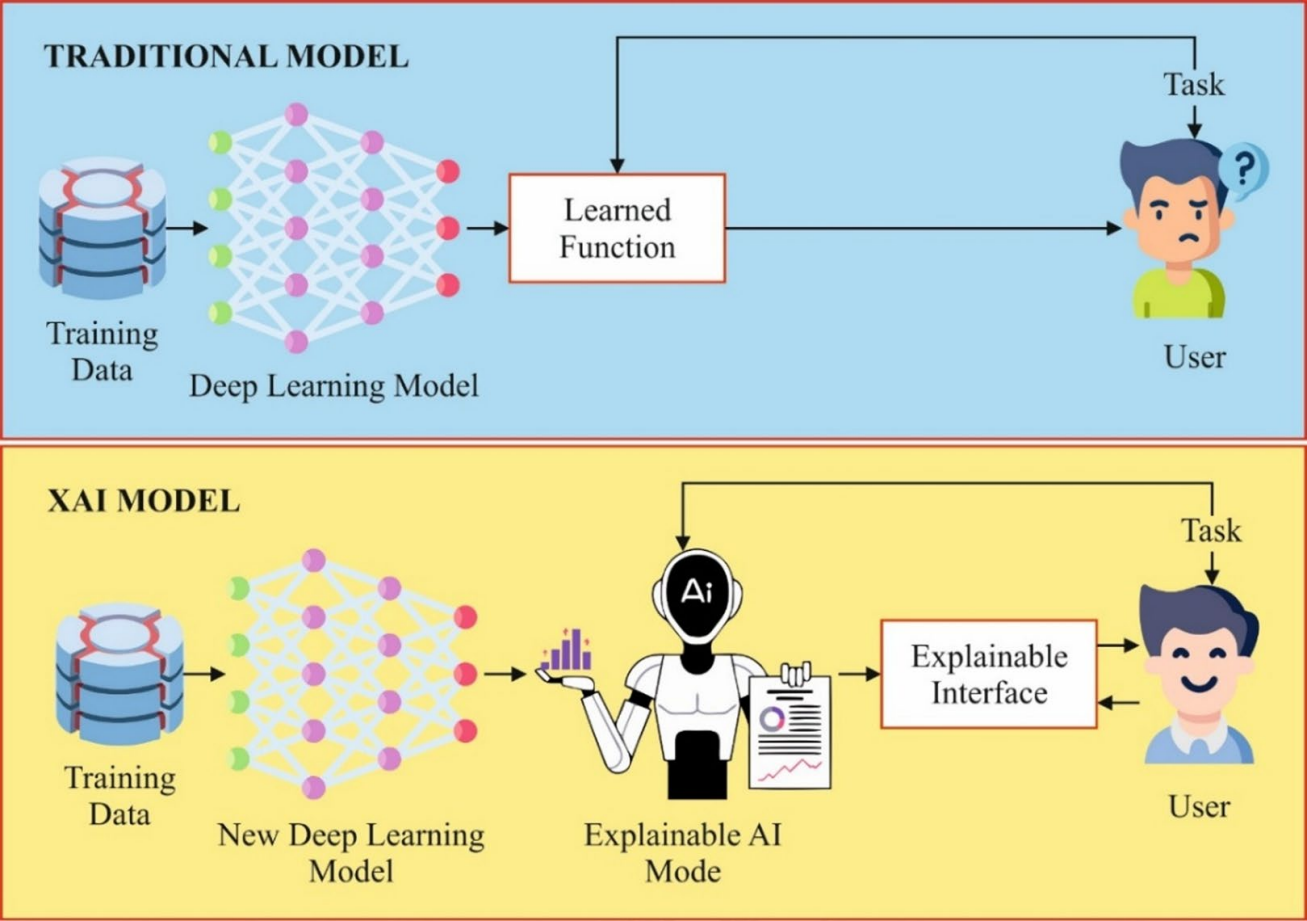
General structure of XAI model.

In recent times, AI is able to synthesise and analyse multimodal data with extraordinary precision and dependability, and has recently been considered for swift development in AI applications in medical domains, such as hepatology. These AI transformations in the last few years are due to the arrival of the deep learning (DL) technique^9^. DL models could process a diverse range of clinical information from structured numeric data: laboratory values and vital signs, higher-dimensional data from multi-omics analyses, and digitalised higher-resolution images from several radiologic analyses^10^. This examination intends to outline and emphasise instances of the numerous DL applications for improving patient care with HCC. Conventional research techniques may be susceptible to biases related to their design, prioritise specific molecules over others, and risk overlooking more efficient biomarkers. Nevertheless, the XAI application presents an effective alternative to conventional techniques. XAI provides a way to accelerate the discovery process, reduce research biases, and decrease expenses, allowing the detection of beneficial insights with higher efficacy and possibly revolutionising the environment of LC treatment. Figure 1 implies the general structure of the XAI model.

This paper presents a Hepatocellular Carcinoma Diagnosis based on an Aggregated Learners Utilising Explainable Artificial Intelligence (HCDAL-XAI) model from biomedical data. The primary purpose of the HCDAL-XAI model is to deliver an accurate detection model for initial diagnosis and efficient treatment of HCC using progressive methods. Initially, the data pre-processing step uses min-max normalisation. Furthermore, the HCDAL-XAI model employs an ensemble of a sparse autoencoder (SAE), gated recurrent unit (GRU), and deep belief network (DBN) for the classification process. Lastly, the explainable AI (XAI) model employs SHapley Additive exPlanations (SHAP) to enhance the reliability of AI methods by making their decision-making processes understandable to humans. The experimental evaluation of the HCDAL-XAI model occurs using a benchmark dataset. The key contributions are listed below.

- Initially, min-max normalisation is applied for scaling the HCC dataset, and the process also ensures that the overall features are in a uniform range, mitigating data variability. The method also improves model convergence and training stability, ultimately resulting in an enhanced classification accuracy and more reliable diagnostic performance.
- A hybrid DL ensemble integrating SAE, GRU, and DBN is employed for effectively capturing both nonlinear and sequential patterns in HCC data. This also improves feature representation, model robustness and presents more accurate and reliable classification outcomes.
- The SHAP-based explainability is employed for making the decision-making process transparent and interpretable, clearly quantifying the impact of each feature, which also assists clinicians in comprehending the reasoning behind predictions and assists in more informed and reliable HCC diagnosis.
- A novel framework is presented by integrating SAE-GRU-DBN ensemble classification, and SHAP-based explainability. This incorporation utilises advanced optimisation algorithms, DL, and XAI techniques in a single pipeline, which improves predictive accuracy, model robustness, and decision transparency.

## Related works

Rhyou and Yoo^11^ introduced HCC-Net, a new wavelet-driven methodology for precise HCC detection from intestinal imagery utilising an artificial neural network (ANN). This methodology merges the discrete wavelet transform (DWT) for image decomposition with a hierarchical cancer detection model to generate pattern-enhanced cancer images. Usha et al.^12^ presented a new technique to classify Fire Hawk Optimiser (FHO) as well as a feature extractor alongside pixelated disparity-enabled Deeper CNN. Though the pixelated disparity-enabled Deep CNN effectively collects intricate data from US imaging, the FHO improves classification efficiency using a learning process optimiser. Gu et al.^13^ intended to design a DL–driven pipeline for automatically segmenting cells in H&E-stained tissue, thus increasing pathological image examination abilities. CSGO architecture incorporates membrane and nuclei segmentation, and post-processing through an energy-based watershed technique. Particularly, utilised the YOLO object detection method to segment nuclei and the U-Net to segment membranes. Rajeev et al.^14^ introduced a novel methodology, the HCCNet Fusion network, a strong incorporation of sophisticated DL methods developed to raise the HCC stage detection precision. Exploiting the interactions among the U-Net and VGG16 frameworks and integrating advanced data pre-processing models: marker-based watershed segmentation and Otsu’s binary thresholding, this model concentrates on reinforcing the HCC stage recognition accuracy. Lin et al.^15^ proposed a hybrid ML model, efficient FS, and an ensemble classifier to detect HCC that is established and relies on the Harris Hawks Optimizer (HHO) procedure. The ensemble classification depends upon the bagging method and is designed using the decision tree technique. Zhang et al.^16^ suggested a new dual-channel attention-sharing hybrid network (DCAH-Net), which depends on multiple feature fusion for automatic HCC grading of histopathology imagery, helping pathologists to diagnose HCC. To resolve the issue that extensive histopathological images can’t be used for training networks straightforwardly, the patch-based system is employed in this study to split extensive images into patches. Transformer network and CNNs are leveraged to extract global and local features within the DCAH-Net. Feng et al.^17^ presented a model for extracting perfusion features in a multi-view learning process by attaining inherent features of LCs, resulting in a deep model to distinguish HCC from other cancerous cases.

Cinar et al.^18^ recommended an innovative HCC classification technique leveraging a hyperspectral image (HS) combined with a light microscope. CNNs with 3D CNNs are utilised to create a precise classification paradigm. Employing 3D CNN, spatial and spectral features in the spectral cube are integrated for training an advanced classifier. Wang et al.^19^ evaluated the efficacy of Xiao-Chai-Hu decoction (XCHD) in treating hepatitis, liver fibrosis, and hepatocellular carcinoma by utilising meta-analysis and five ML models, namely Random Forest (RF), Extreme Gradient Boosting (XGBoost), Lasso, Multilayer Perceptron (MLP), and a stacking model. Hu et al.^20^ evaluated a novel biotransformation-integrated network pharmacology strategy by integrating absorption, distribution, metabolism, excretion, and toxicity (ADMET) analysis, molecular docking, molecular dynamics simulation, and experimental validation in cells and animals. Men et al.^21^ aimed to summarise the development and applications of activatable fluorescent probes for the detection and imaging of hepatocellular carcinoma. Li et al.^22^ developed an adaptive mini-minimum spanning tree-based outlier detection (MMOD) method to detect outliers in medical and other datasets by using a scaled Euclidean distance measure. Savaş^23^ developed a comprehensive DL methodology integrating stacked ensemble learning, multi-task learning, transfer learning (TL), and XAI to diagnose automatically and stage liver cirrhosis using T2-weighted MRI images. Zarlashat, Tayyeba, and Hussain^24^ evaluated the prognostic value of inflammatory markers, specifically the neutrophil-to-lymphocyte ratio (NLR) and platelet-to-lymphocyte ratio (PLR), in hepatocellular carcinoma. Tang et al.^25^ developed an interpretable ML method by utilising XGBoost incorporated with least absolute shrinkage and selection operator regression, RF, and recursive feature elimination. Zhang et al.^26^ developed early hepatocellular carcinoma predictors (eHCC-pred) by utilising ML-based methods with advanced FS. Peng et al.^27^ developed a 3-D convolutional block attention module (CBAM) AI model for accurate hepatocellular carcinoma detection using noncontrast computed tomography scans. Li et al.^28^ developed a non-invasive auxiliary assessment method using computed tomography-derived extracellular volume (CT-ECV) and six ML models, such as logistic regression (LR), XGBoost, Light Gradient Boosting Machine (LightGBM), RF, adaptive boosting (AdaBoost), and Gaussian naive Bayes (GNB) for predicting the HCC.

### Limitations of the existing studies and research gap

Though the existing methods are effective, they show various limitations, as HCC-Net, FHO-DCNN, CSGO pipeline, HCCNet Fusion, HHO-ensemble, DCAH-Net, multi-view learning, and 3D CNN hyperspectral methods rely on complex pre-processing or extensive manual annotation, restricting scalability. Also, a few methods concentrate on particular image models, mitigating generalisation across datasets. Furthermore, it is challenging to handle large histopathological images, and restrictions can be seen in integrating multimodal data and improving the interpretability of ANN and CNN-based models.

- The research gap is in developing a robust, generalised, and fully automated HCC detection framework that addresses data heterogeneity, computational efficiency, and interpretability while maintaining high accuracy.
- Various studies concentrate on either imaging-based or biomarker-based HCC detection, but few integrate multimodal data (e.g., hyperspectral, MRI, CT, and biochemical markers) for comprehensive diagnosis.
- Diverse DL and ML methods lack interpretability, restricting clinical trust and adoption in real-world settings.
- Existing approaches mostly depend on small or single-center datasets, raising concerns about generalizability and robustness across various patient populations.
- Early-stage HCC prediction or real-time screening tools are addressed by the research, appropriate for routine clinical workflows.
- Few studies incorporate outlier detection, class imbalance handling, or uncertainty estimation, which are critical for reliable performance in heterogeneous clinical datasets.

## Materials and methods

**Fig. 2:**
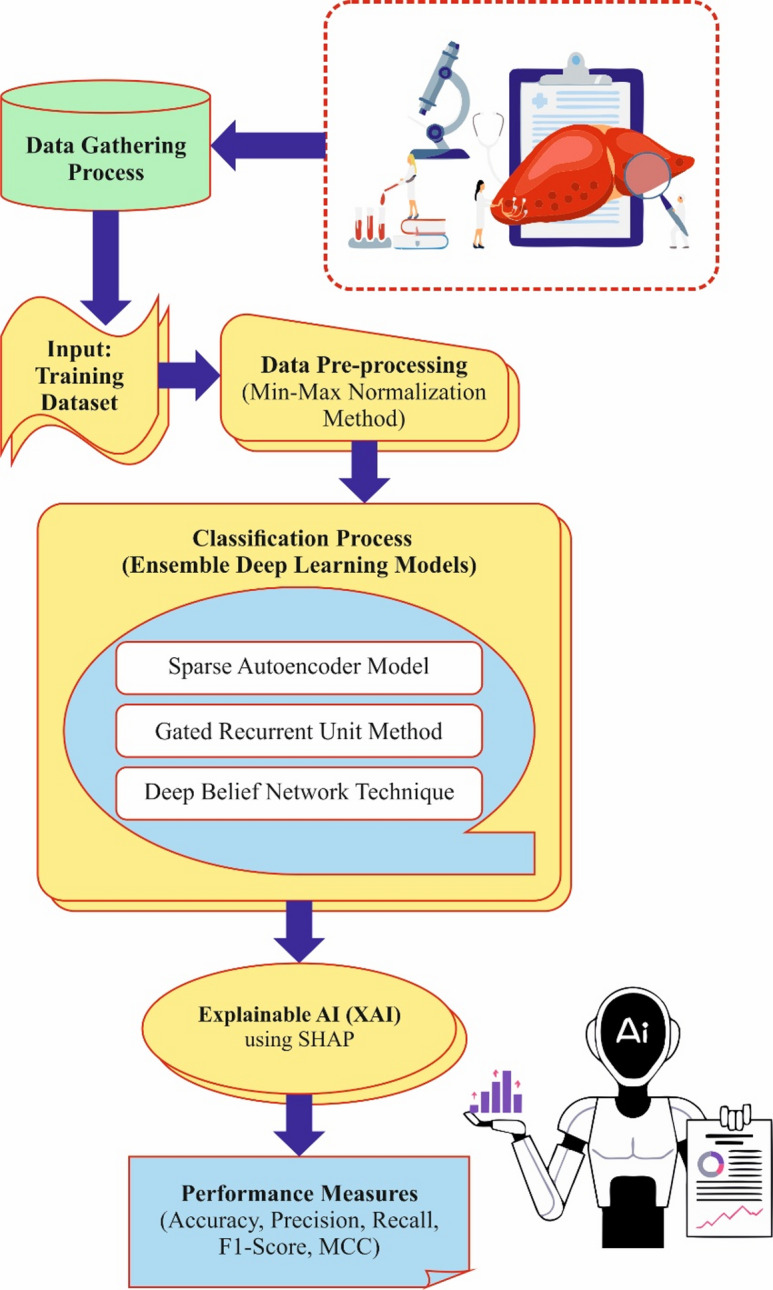
Complete process of HCDAL-XAI methodology.

In this manuscript, the HCDAL-XAI approach is proposed. The primary purpose of the HCDAL-XAI model is to deliver an accurate detection model for initial diagnosis and efficient treatment of HCC using progressive methods. It encompasses data pre-processing, dimensionality reduction, ensemble classification, and XAI techniques. Figure 2 illustrates the complete process of the HCDAL-XAI model.

### Stage 1: data standardisation

Primarily, the data pre-processing step uses min-max normalisation for altering input data into a useful pattern^29^. This process proves the convergence speed of learning algorithms and preserves the original data distribution without changing the relationships between values, compared to standardisation or z-score normalisation. This is also easy to implement and is compatible with optimisation algorithms, ensuring faster and more stable training. Moreover, this mitigates the risk of numerical instability in activation functions such as sigmoid or tanh. Thus, this provides a balanced, effective pre-processing step that improves model accuracy and reliability for HCC classification.

Normalising the scale of the data is needed to avoid these problems. Using a numerical function, mathematical values are converted into a new area in the usage of the Min-Max Normalisation model. The dataset is standardised utilising this model in this proposed technical study. The min‐max normalisation is the standard method for data normalisation.

The dataset values are standardised for values inside ranges (max. and min. values from datasets), utilising the formulation below. These standardised data are scaled to ranges of $\:\left[\mathrm{0,1}\right]$ or [−1, 1] for input values $\:x$ of feature $\:X$ to $\:xnorm$ in ranges $\:[low,\:high]$.

1 $$\:\chi \:_{{norm}} = \frac{{(high - low) \cdot \:(x - \:\min \:X)}}{{\:\max \:X - \:\min \:X}}$$

Whereas $\:minX$ and $\:maxX$ are the min and max values of the input data feature $\:X.$ Therefore, the scaled data in the interval of $\:\left[\mathrm{0,1}\right]$ are directed for FS.

### Stage III: model selection for ensemble classification process

In this stage, the HCDAL-XAI model employs an ensemble of SAE, the GRU, and DBN for the classification process^30^. The SAE efficiently learns sparse and meaningful feature representations, while sequential dependencies are effectively captured by GRU. Also, DBN models intrinsic nonlinear relationships. The ensemble model also mitigates bias and enhances generalisation and robustness against overfitting, compared to utilising a single classifier, making it particularly effective for heterogeneous HCC data. Furthermore, a weighted voting mechanism is employed for aggregating the outputs of the three models, where the prediction of each model contributes proportionally to its performance or confidence score. The overall performance is also improved by this aggregation and by integrating complementary strengths of each classifier, smoothing out individual errors, and producing a more reliable final prediction. Thus, the ensemble ensures higher accuracy and stability compared to standalone models. A meta-classifier is used for combining the outputs of the ensemble model, which learns to optimally integrate predictions for accurate HCC diagnosis.

#### SAE method

**Fig. 3:**
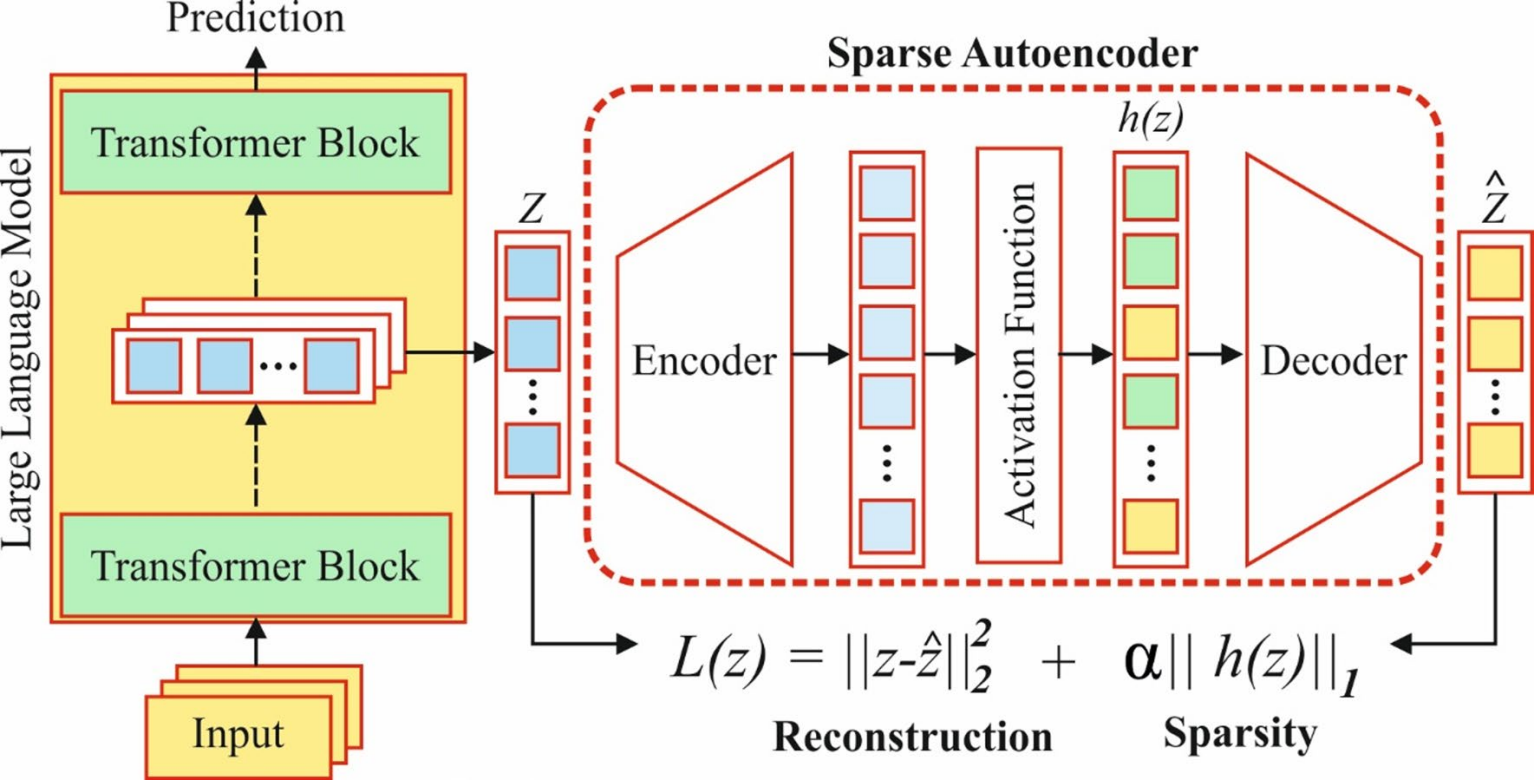
Structure of the SAE method.

An autoencoder (AE) is a form of NN that uses a backpropagation (BP) model to attain output values that are equivalent to the input values^31^. The encoder transforms the input into a concealed representation, whereas the decoder tries to rebuild the new input by mapping these latent representations. The primary goal of the model is to learn a function, $\:hw,b\left(x\right)\approx\:x$, while gaining lower-dimensional representations of the input data. The final aim is to represent the original data in a small size with minimum information loss, with the critical feature that the counts of nodes in the input layer (eliminating bias nodes) are equivalent to the node counts in the output layer. In contrast, the node counts in the hidden layers (HLs) are lower. Figure 3 specifies the structure of the SAE method.

While the number of nodes within the HL is larger than the number of nodes in the input layer, the self-coding is even used, but in addition to a sparsity restriction. This guarantees that the majority of nodes in the HL are suppressed, and only a smaller part is started, as a result attaining a similar effect. This kind of AE is well-known as the sparse self‐encoder. The data for the average activation of the sparse auto‐coding HL is signified as

2 $$\:{\widehat{\rho\:}}_{\mathrm{j}}=\frac{1}{m}{\sum\:}_{i=1}^{j}\left\{{a}_{j}^{\left(2\right)}\left[{x}^{\left(i\right)}\right]\right\}$$.

Here $\:{a}_{j}^{\left(2\right)}\left(x\right)$ denotes the activation degree of the hidden neuron $\:j$ after the input data is $\:x.$ To create the mean activation near a somewhat smaller value of $\:p$, the relative entropy of $\:p$ and $\:p$ is presented as the penalty term, and the succeeding loss function is obtained.

#### GRU technique

GRU is one of the enhanced versions of recurrent neural networks (RNN)^32^. In comparison to conventional RNNs, the GRU effectively mitigates vanishing gradient in longer-sequence training over reset and update gates, although preserving lower computation complexity. The computational procedure is described.

(1) The update gate $\:{z}_{t}$ regulates the proportion of preceding HL data transferred to the existing moment:

3 $$\:{z}_{t}=\sigma\:\left({W}_{z}{x}_{t}+{U}_{z}{h}_{t-1}\right)$$

Now $\:{W}_{z}$ and $\:{U}_{z}$ refer to the weighted matrices of the update gate, $\:\sigma\:$ indicates the activation function of the sigmoid, $\:{h}_{t-1}$ represents the preceding HL, and $\:{x}_{t}$ depicts the existing input.

(2) The resetting gate $\:{r}_{t}$ controls the impact of historic state data on existing candidate HL:

4 $$\:{r}_{t}=\sigma\:\left({W}_{r}{x}_{t}+{U}_{r}{h}_{t-1}\right)$$

Here, $\:{W}_{r}$ and $\:{U}_{r}$ refer to the reset gate’s weighted matrices.

(3) The candidate HL $\:{h}_{t}$ integrates present input and controlled historical data:

5 $$\:{\stackrel{\sim}{h}}_{t}=\mathrm{t}\mathrm{a}\mathrm{n}\mathrm{h}\left(W{x}_{t}+U\left({r}_{t}\odot\:{h}_{t-1}\right)\right)$$

Now $\:W$ and $\:U$ represent weighted matrices for landmark data.

(4) The final HL is attained through linearly interpolating the candidate HL and preceding HL over the upgrade gate:

6 $$\:{h}_{t}=\left(1-{z}_{t}\right)\odot\:{h}_{t-1}+{z}_{t}\odot\:{\stackrel{\sim}{h}}_{t}$$

**Fig. 4:**
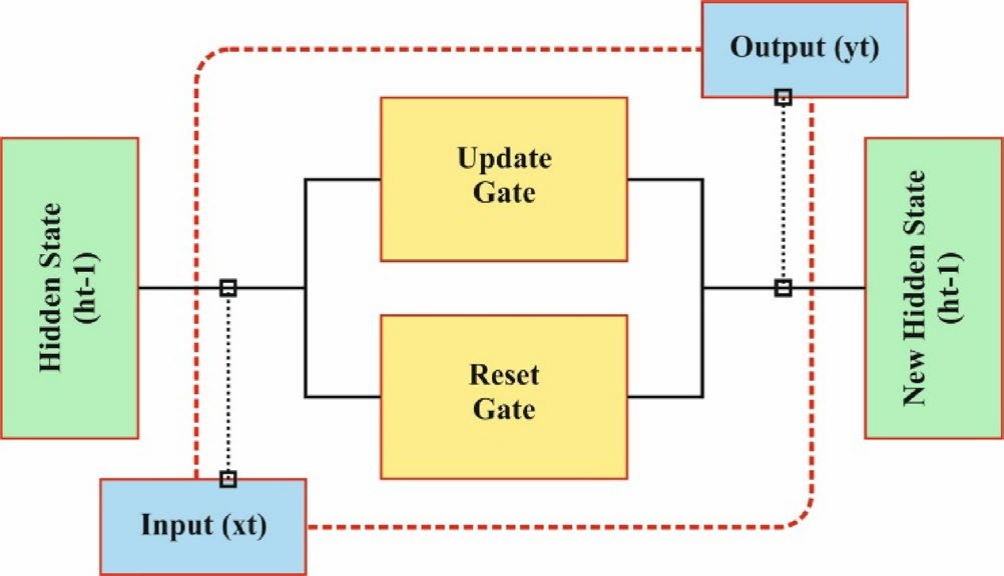
Framework of GRU.

While either LSTM or GRU depends upon the RNN framework, they vary substantially in structural patterns. The GRU accepts a more simplified framework with merely dual-gated components, such as the reset gate and update gate, contrasted with the three gates, namely forget, output, and input in LSTM. Figure 4 indicates the framework of the GRU methodology.

#### DBN model

DBN is a generative technique that works by stacking restricted Boltzmann machines (RBM) layer by layer to remove substantial aspects from diagnostic data or clinical records^33^. The method learns the joint probability distribution through every visible layer (VL) and HL. The DBN might be depicted by the succeeding joint probability in Eq. (7).

7 $$\:o(\gamma \:,\:g^{1} ,\:g^{2} ,\:...g^{m} ) = o(\gamma \:|g^{1} )o(g^{1} |g^{2} )\: \ldots o\left( {g^{{m - 1}} |g^{m} } \right)$$

Now $\:{g}^{1},$ $\:{g}^{2},\dots\:\dots\:,$ and $\:{g}^{m}$ refer to HL, and $\:\gamma\:$ depicts VL. The conditional distribution among HL is expressed by $\:o\left({g}^{m-1}\right|{g}^{m})$. Every RBM is described by VL $\:d=(d1,\:d2,\:\dots\:dn)$ and HL $\:=$ $\:({g}_{1},\:{g}_{2},\dots\:\dots\:,\:{g}_{k})$, where the energy function $\:F(\gamma\:,\:g;\theta\:)$ was employed to assess the state of the system in Eq. (8).

8 $$\:F\left(\gamma\:,\:g;\theta\:\right)=-{\sum\:}_{j=1}^{k}{\sum\:}_{i=1}^{n}{\omega\:}_{ji}{g}_{j}{\gamma\:}_{i}-{\sum\:}_{i=1}^{n}{a}_{i}{\gamma\:}_{i}-{\sum\:}_{j=1}^{k}{d}_{j}{g}_{j}$$

Here, $\:{d}_{j},$ $\:{a}_{i}$ refers to biases, and $\:{\omega\:}_{ji}$ represents the weight among HL and VL. To compute the conditional probability of VL and HL in Eqs. (9) & (10).

9 $$\:o\left({\gamma\:}_{i}|g;\theta\:\right)=\sigma\:\left({\sum\:}_{j=1}^{k}{\omega\:}_{ji}{g}_{j}+{d}_{j}\right)$$

10 $$\:o\left({g}_{j}|\gamma\:;\theta\:\right)=\sigma\:\left({\sum\:}_{i=1}^{n}{\omega\:}_{ji}{\gamma\:}_{j}+{a}_{i}\right)$$

**Fig. 5:**
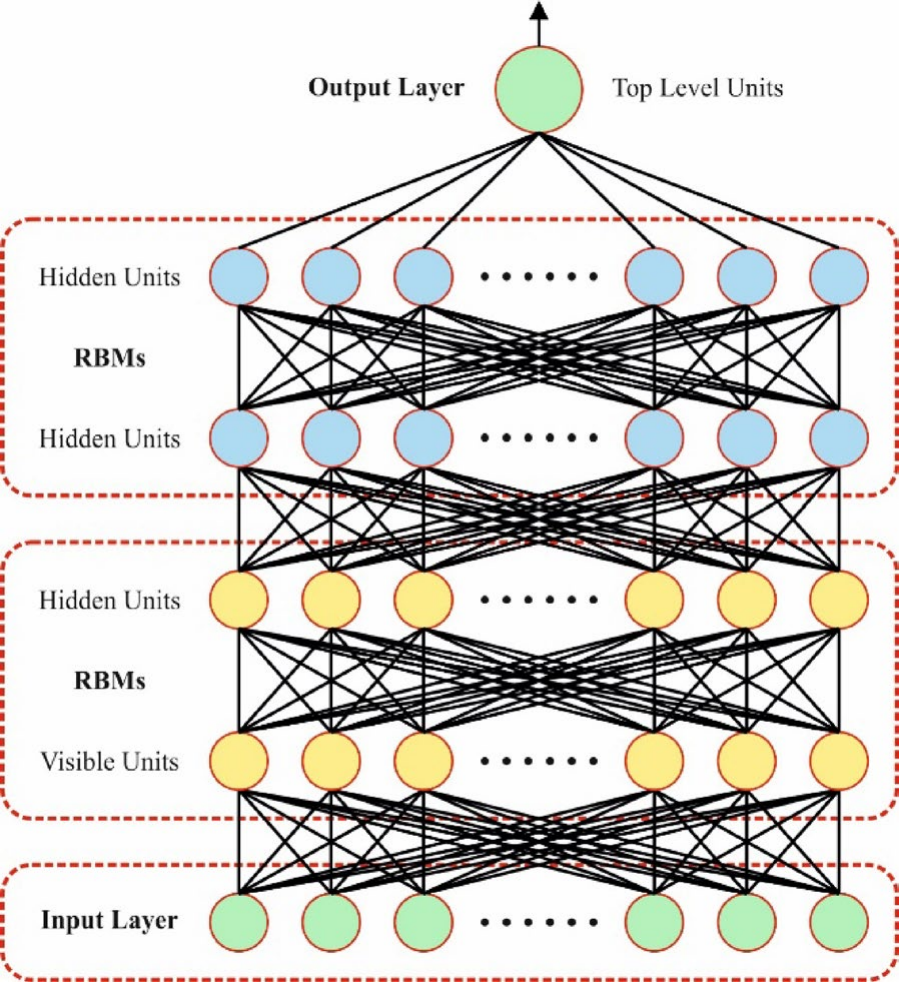
Architecture of the DBN method.

Now $\:\sigma\:\left(w\right)=1/(1+\mathrm{e}\mathrm{x}\mathrm{p}(-w\left)\right)\:$ represents the sigmoid function. The learning procedure for RBM comprises an upgrade employing contrastive divergence, which is depicted in Eqs. (11, 12 and 13). Figure 5 illustrates the architecture of the DBN approach.

11 $$\:{\omega\:}_{ji}^{\left(s\right)}\leftarrow\:{\omega\:}_{ji}^{(s-1)}+\eta\:\left(o\left({g}_{j}|{\gamma\:}_{j};\theta\:\right){\gamma\:}_{j}-o\left({g}_{j+1}|{\gamma\:}_{j+1};\theta\:\right){\gamma\:}_{j+1}\right)$$

12 $$\:{d}_{j}\leftarrow\:{d}_{j-1}+\eta\:\left({g}_{j}-{g}_{j+1}\right)$$

13 $$\:{a}_{i}\leftarrow\:{a}_{i-1}+\eta\:\left({\gamma\:}_{j}-{\gamma\:}_{j+1}\right)$$

Table 1 describes the hyperparameters involved in the ensemble model.

**Table 1:** Hyperparameters of the ensemble SAE, GRU, and DBN models.

Model	Key Hyperparameters	Typical Values/Notes
SAE	INPUT_SIZE	Relies on dataset features
	HIDDEN_LAYERS	1–3 layers
	ACTIVATION	ReLU/Sigmoid
	SPARSITY_PARAM ($\:\rho\:$)	0.05–0.1
	LEAR_RATE	0.001–0.01
	EPOCHS	50–200
GRU	INPUT_SIZE	Feature size from SAE or raw data
	HIDDEN_UNITS	32–128
	LAYERS	1–3
	DROPOUT	0.2–0.5
	LEAR_RATE	0.001–0.01
	EPOCHS	50–150
DBN	RBM_LAYER_NO	2–4
	HIDDEN_UNITS_PER_RBM	64–256
	LEAR_RATE	0.001–0.01
	EPOCHS_PER_RBM	50–100
	FINE_TUNING	Backpropagation with softmax/classifier layer

### Stage V: modelling of XAI using SHAP

Finally, the SHAP is used for the XAI model to improve the clarity of AI techniques^34^. The clarity and transparency of AI-based predictions are improved by SHAP, and it also ensures consistent and fair attribution of feature importance across predictions. The technique also provides both global and local explanations with higher consistency and reliability, and compared to other explainability techniques such as LIME or feature importance scores, SHAP allows seamless integration with complex ensemble models like SAE–GRU–DBN. SHAP explanations are intuitive and visually interpretable, which supports clinician trust and understanding.

XAI is intended to make a type of tools, methods or approaches for producing more explainable, transparent, and responsible systems, while maintaining its effective outcomes. For critical decision-making, it is essential to understand the rationale behind how the system generates major decisions. Therefore, the significance of the XAI technique is now growing into a strong one. Moreover, the black-box type of the AI-based model provides tremendous outcomes and also includes no explanations. Later, it loses its reliance on accepting these models in significant decision-making. Numerous XAI techniques, such as the SHAP architecture, are one type of technique. SHAP is an explainable ML algorithm that is utilised for local and global explanations. The SHAP approach measures the involvement of every single feature in the model prediction. During local explanations, SHAP computes the baseline values and the influence of all the features, Eq. (15), to describe the outcome. The computation of the evaluation of feature $\:i$ to sample $\:x$, alternatively called the SHAP evaluation, can be appropriately calculated as given in Eq. (14).

14 $$\:{\varphi\:}_{i}={\sum\:}_{S\subseteq\:F\setminus\:\left\{i\right\}}^{\:}\frac{\left|S\right|!\left(\right|F|-|S|-1)!}{\left|F\right|!}\left[{f}_{S\cup\:\left\{i\right\}}\left({x}_{s\cup\:\left\{i\right\}}\right)-{f}_{s}\left({x}_{s}\right)\right]$$

15 $$\:f\left(x\right)={\varphi\:}_{0}+\sum\:{\varphi\:}_{i}$$

The primary intention is to find sample $\:x$ as explained, along with the model prediction $\:f\left(x\right)$. The following stages include measuring the baseline prediction $\:{\varphi\:}_{0}$, Eq. (15), which can be predicted when no features have been identified. It is vital to assess every feature category $\:S\subseteq\:F$ for evaluating the modification in the forecast while the feature $\:i$ will be included. The minimal contribution of all features is estimated as the dissimilarity between$\:\:f\left(S\right)$ and$\:\:f\left(SU\right\{i\left\}\right)$. The minimum involvement is averaged by applying weights that depend on Shapley theory, as shown in Eq. (14). The model prediction, represented by $\:\left(x\right)$, is well-described as the sum of the baseline prediction and the entire feature contribution values, as specified in Eq. (15). The high SHAP importance of clinical features such as Albumin and Ferritin aligns with existing hepatology literature, in which albumin highlights liver synthetic function and ferritin shows inflammation and iron overload, both is firmly associated with HCC progression, thereby assisting the clinical validity beyond predictive accuracy.

## Results evaluation and discussion

The simulation validation of the HCDAL-XAI model is studied under the HCC dataset [https://www.kaggle.com/datasets/mrsantos/hcc-dataset, 35]. The model is simulated using Python 3.6.5 on a PC with an i5-8600k, 250GB SSD, GeForce 1050Ti 4GB, 16GB RAM, and 1 TB HDD. Parameters include a learning rate of 0.01, ReLU activation, 50 epochs, 0.5 dropout, and a batch size of 5.

This dataset comprises 165 samples under dual patient labels, like dies and lives. The complete details of this dataset are exhibited below in Table 2. The no. of features is 49, but only 30 features are chosen. The dataset covers a 1-year follow-up period to assess patient survival outcomes.This dataset provides a clear and balanced representation of HCC patient outcomes, making it appropriate for predictive modelling. The class-weighted learning and robust evaluation metrics are utilised for handling class imbalance and overfitting. Also, the missing data is efficiently handled by utilizing statistical imputation (e.g., mean/median imputation), followed by normalization for ensuring data completeness and stable model training. Thus, generalisation is improved through these strategies by enabling the model to learn meaningful patterns and maintain stable performance on unseen data.

**Table 2:** Details of the dataset.

Patients Label	No. of Samples
Dies (0)	63
Lives (1)	102
Total Samples	165

**Fig. 6:**
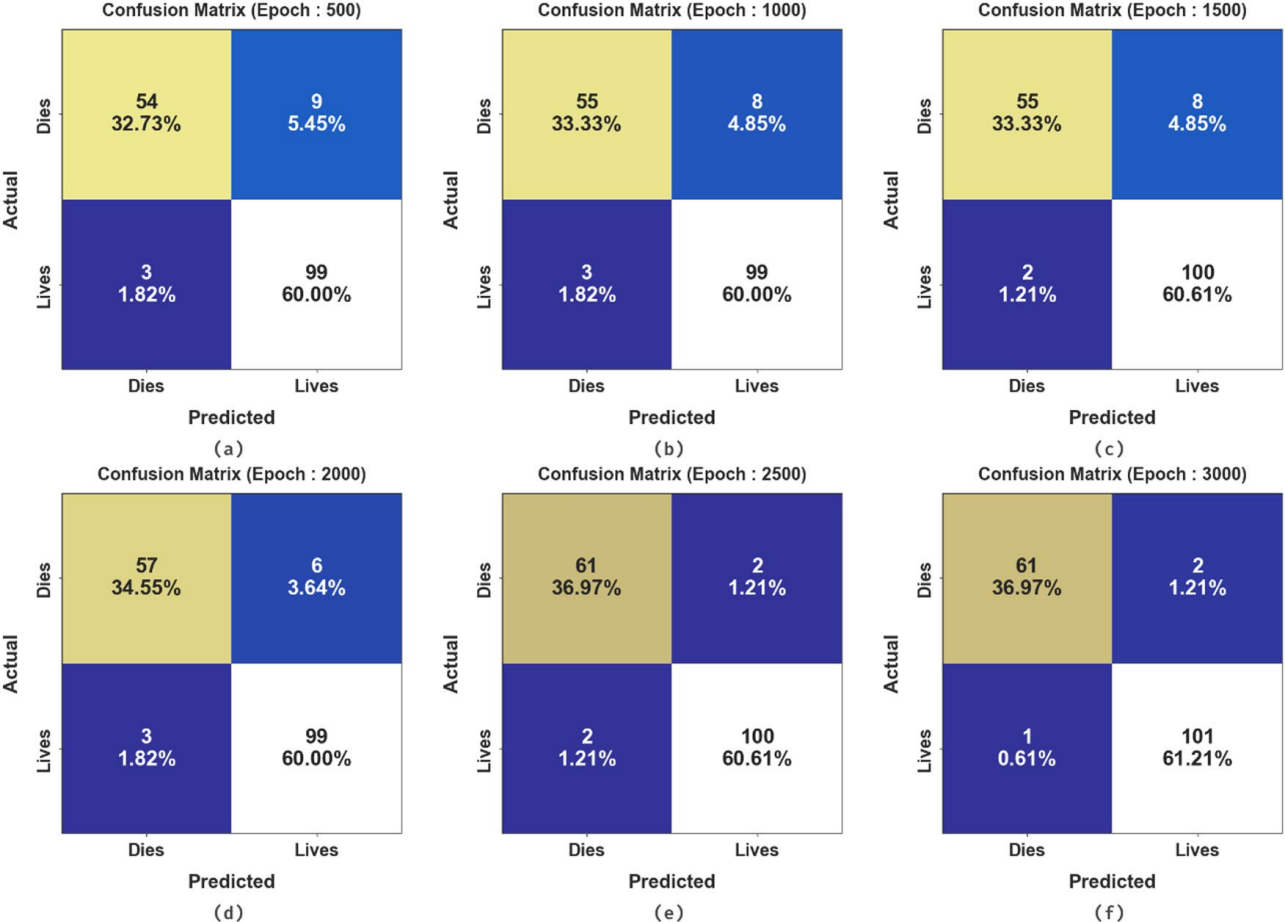
Confusion matrices of HCDAL-XAI model (**a**-**f**) 500–3000 Epochs.

Figure 6 presents the confusion matrices generated by the HCDAL-XAI approach on numerous epochs. The results state that the HCDAL-XAI method effectively recognises each class.

**Fig. 7:**
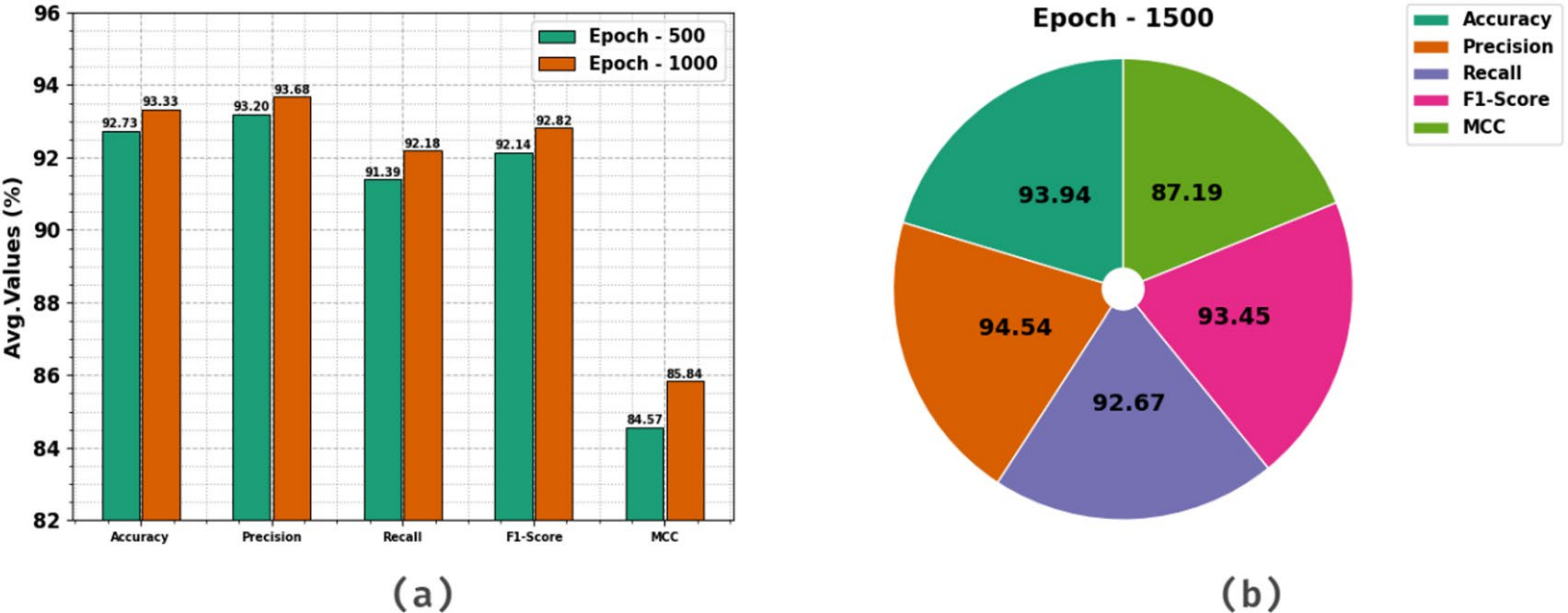
Average values of HCDAL-XAI method under (**a**-**b**) Epochs 500–1000, and (b) Epoch 1500.

Table 3 exemplifies the HCC detection of the HCDAL-XAI method at distinct epochs. The HCC detection of the HCDAL-XAI method is established at Epochs 500–1500 in Fig. 7. Under 500 epochs, the HCDAL-XAI method acquires an average $\:acc{u}_{y}$, $\:pre{c}_{n}$, $\:rec{a}_{l}$, $\:F{1}_{score},\:$and $\:MCC$ of 92.73%, 93.20%, 91.39%, 92.14%, and 84.57%. Moreover, under 1000 epochs, the HCDAL-XAI method obtains an average $\:acc{u}_{y}$, $\:pre{c}_{n}$, $\:rec{a}_{l}$, $\:F{1}_{score},\:$and $\:MCC$ of 93.33%, 93.68%, 92.18%, 92.82%, and 85.84%. Similarly, at 1500 epochs, the HCDAL-XAI model obtains an average $\:acc{u}_{y}$, $\:pre{c}_{n}$, $\:rec{a}_{l}$, $\:F{1}_{score},\:$and $\:MCC$ of 93.94%, 94.54%, 92.67%, 93.45%, and 87.19%.

**Table 3:** HCC recognition of HCDAL-XAI method under diverse epochs.

Class Labels	$\:\boldsymbol{A}\boldsymbol{c}\boldsymbol{c}{\boldsymbol{u}}_{\boldsymbol{y}}$	$\:\boldsymbol{P}\boldsymbol{r}\boldsymbol{e}{\boldsymbol{c}}_{\boldsymbol{n}}$	$\:\boldsymbol{R}\boldsymbol{e}\boldsymbol{c}{\boldsymbol{a}}_{\boldsymbol{l}}$	$\:{\boldsymbol{F}1}_{\boldsymbol{s}\boldsymbol{c}\boldsymbol{o}\boldsymbol{r}\boldsymbol{e}}$	$\:\boldsymbol{M}\boldsymbol{C}\boldsymbol{C}$
Epoch − 500					
Dies	92.73	94.74	85.71	90.00	84.57
Lives	92.73	91.67	97.06	94.29	84.57
Average	92.73	93.20	91.39	92.14	84.57
Epoch − 1000					
Dies	93.33	94.83	87.30	90.91	85.84
Lives	93.33	92.52	97.06	94.74	85.84
Average	93.33	93.68	92.18	92.82	85.84
Epoch − 1500					
Dies	93.94	96.49	87.30	91.67	87.19
Lives	93.94	92.59	98.04	95.24	87.19
Average	93.94	94.54	92.67	93.45	87.19
Epoch − 2000					
Dies	94.55	95.00	90.48	92.68	88.41
Lives	94.55	94.29	97.06	95.65	88.41
Average	94.55	94.64	93.77	94.17	88.41
Epoch − 2500					
Dies	97.58	96.83	96.83	96.83	94.86
Lives	97.58	98.04	98.04	98.04	94.86
Average	97.58	97.43	97.43	97.43	94.86
Epoch − 3000					
Dies	98.18	98.39	96.83	97.60	96.14
Lives	98.18	98.06	99.02	98.54	96.14
Average	98.18	98.22	97.92	98.07	96.14

**Fig. 8:**
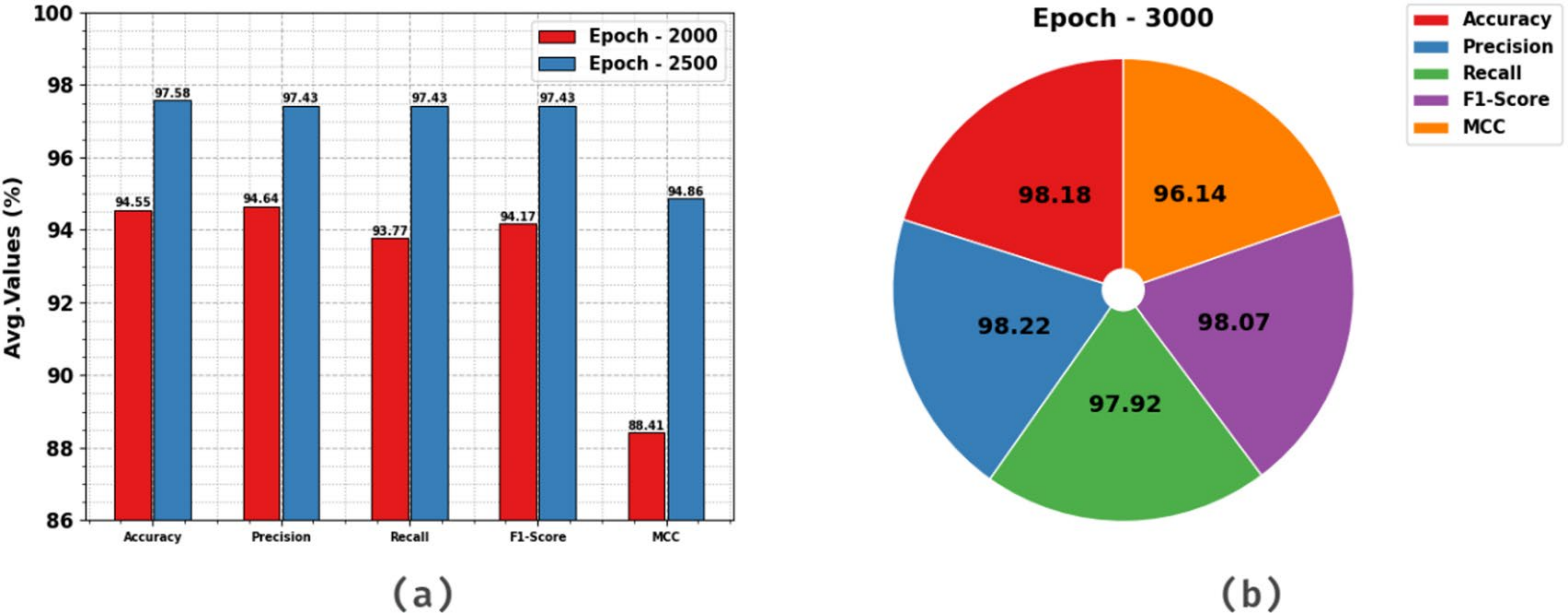
Average values of HCDAL-XAI approach under (**a**-**b**) Epochs 2000–2500, and (b) Epoch 3000.

Figure 8 depicts the HCC detection of the HCDAL-XAI approach on Epochs 2000–3000. Under 2000 epochs, the HCDAL-XAI model gets an average $\:acc{u}_{y}$, $\:pre{c}_{n}$, $\:rec{a}_{l}$, $\:F{1}_{score},\:$ and $\:MCC$ of 94.55%, 94.64%, 93.77%, 94.17%, and 88.41%. Moreover, on 2500 epochs, the HCDAL-XAI model attains an average $\:acc{u}_{y}$, $\:pre{c}_{n}$, $\:rec{a}_{l}$, $\:F{1}_{score},\:$ and $\:MCC$ of 97.58%, 97.43%, 97.43%, 97.43%, and 94.86%. Similarly, at 3000 epochs, the HCDAL-XAI model gets an average $\:acc{u}_{y}$, $\:pre{c}_{n}$, $\:rec{a}_{l}$, $\:F{1}_{score},\:$ and $\:MCC$ of 98.18%, 98.22%, 97.92%, 98.07%, and 96.14%.

**Fig. 9:**
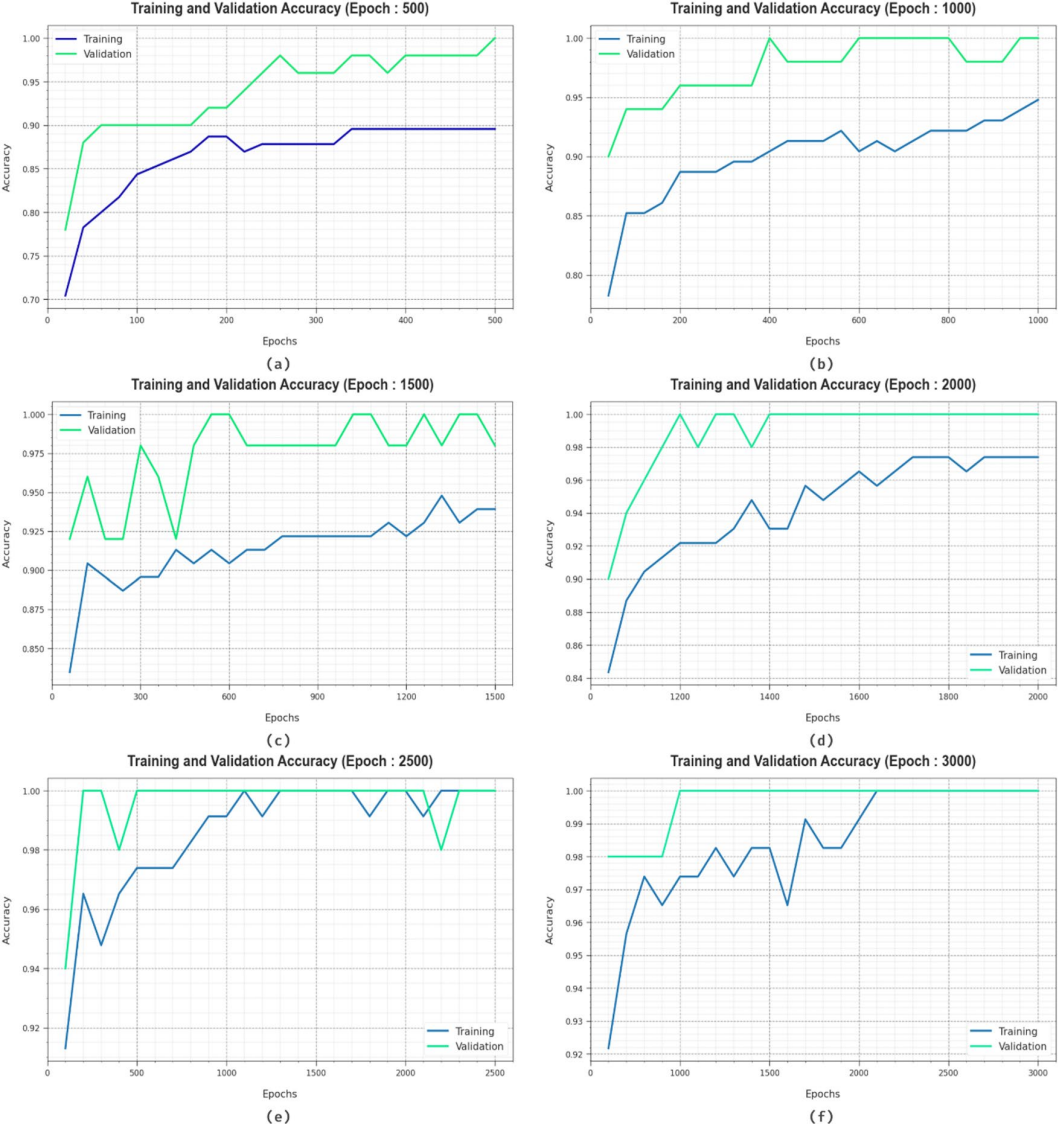
$\:Acc{u}_{y}$ curve of HCDAL-XAI model under (**a**-**f**) 500–3000 Epochs.

In Fig. 9, the training (TRAN) $\:acc{u}_{y}$ and validation (VALD) $\:acc{u}_{y}$ results of the HCDAL-XAI approach at several epochs are depicted. The figure indicates that the TRAN and VALD $\:acc{u}_{y}$ values demonstrate upward tendencies, indicating the efficiency of the HCDAL-XAI approach with significant performance across multiple iterations. The TRAN and VALD $\:acc{u}_{y}$ stay close throughout the epochs, denoting minimal overfitting and demonstrating excellent outcomes of the HCDAL-XAI model, promising stable prediction on unnoticed instances.

**Fig. 10:**
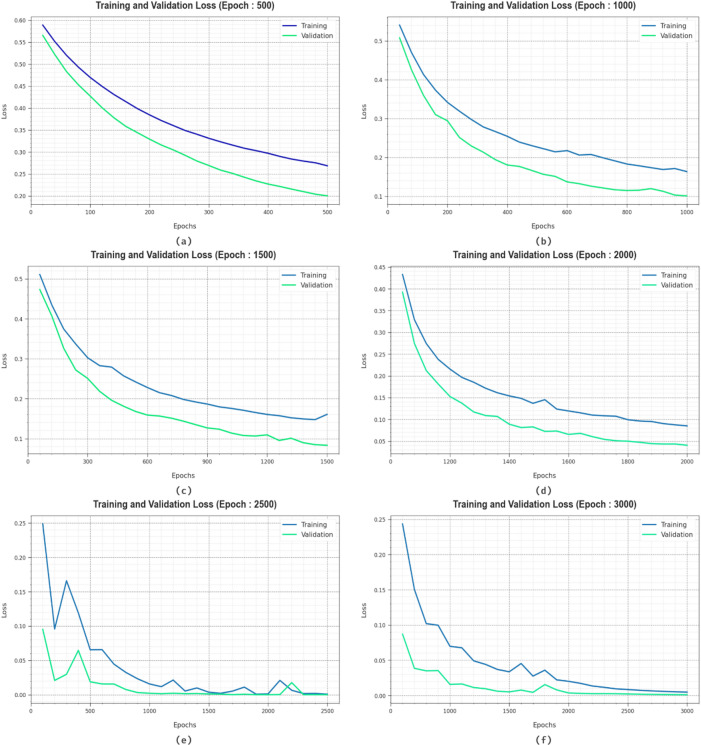
Loss curve of HCDAL-XAI model under (**a**-**f**) 500–3000 Epochs.

In Fig. 10, the TRAN and VALD loss graphs of the HCDAL-XAI model on various epochs are portrayed. It is shown that the TRAN and VALD values elucidate downward tendencies, describing the proficiency of the HCDAL-XAI model to balance a trade-off between data fitting and generality. The persistent drop further promises a better outcome for the HCDAL-XAI method and eventually adjusts the prediction performances.

**Fig. 11:**
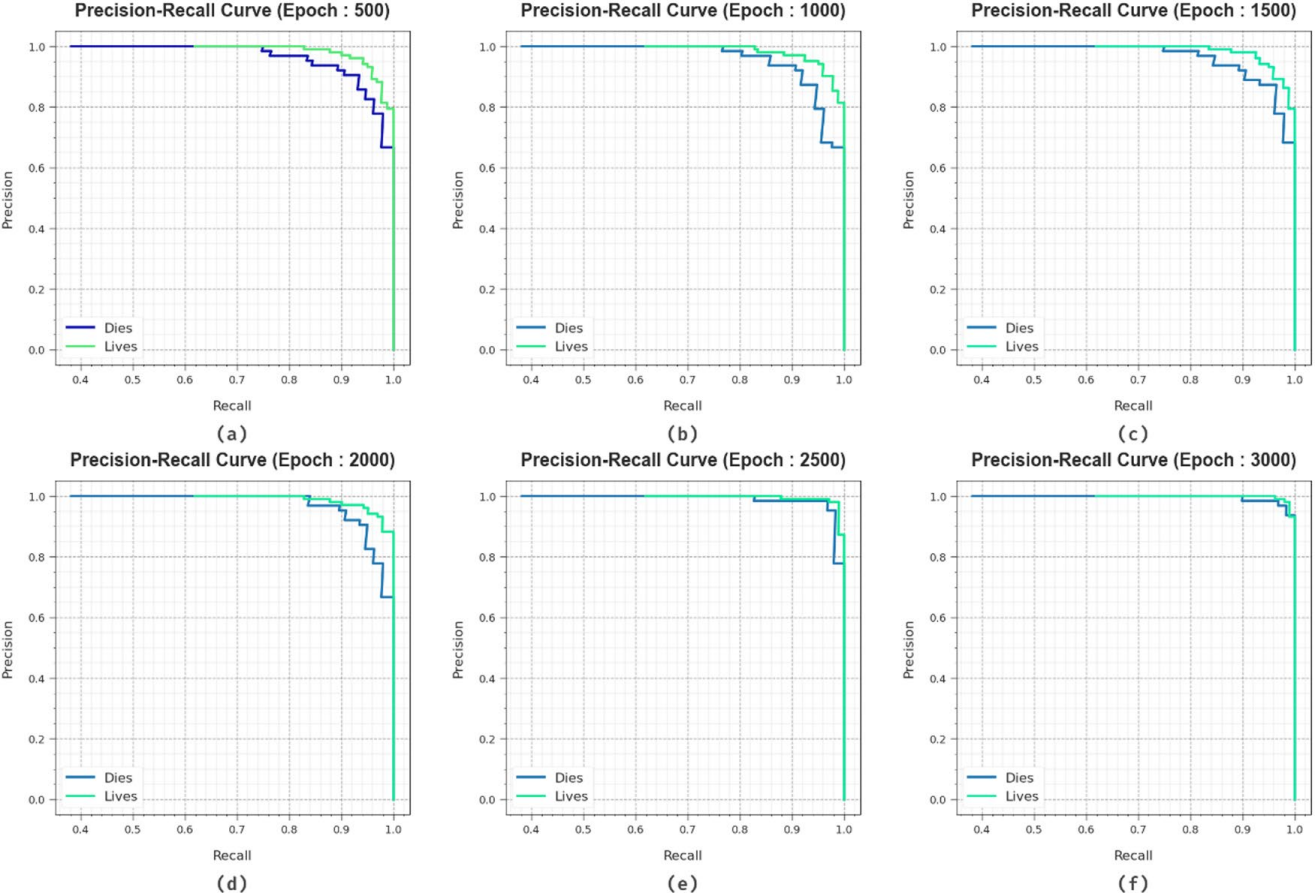
PR curve of HCDAL-XAI method under (**a**-**f**) 500–3000 Epochs.

In Fig. 11, the PR curve inspection of the HCDAL-XAI method at several epochs delivers insights into its solution by mapping Precision against Recall for every class. The persistent increase in PR performances in every label showcases the proficiency of the HCDAL-XAI model during the classification stage.

**Fig. 12:**
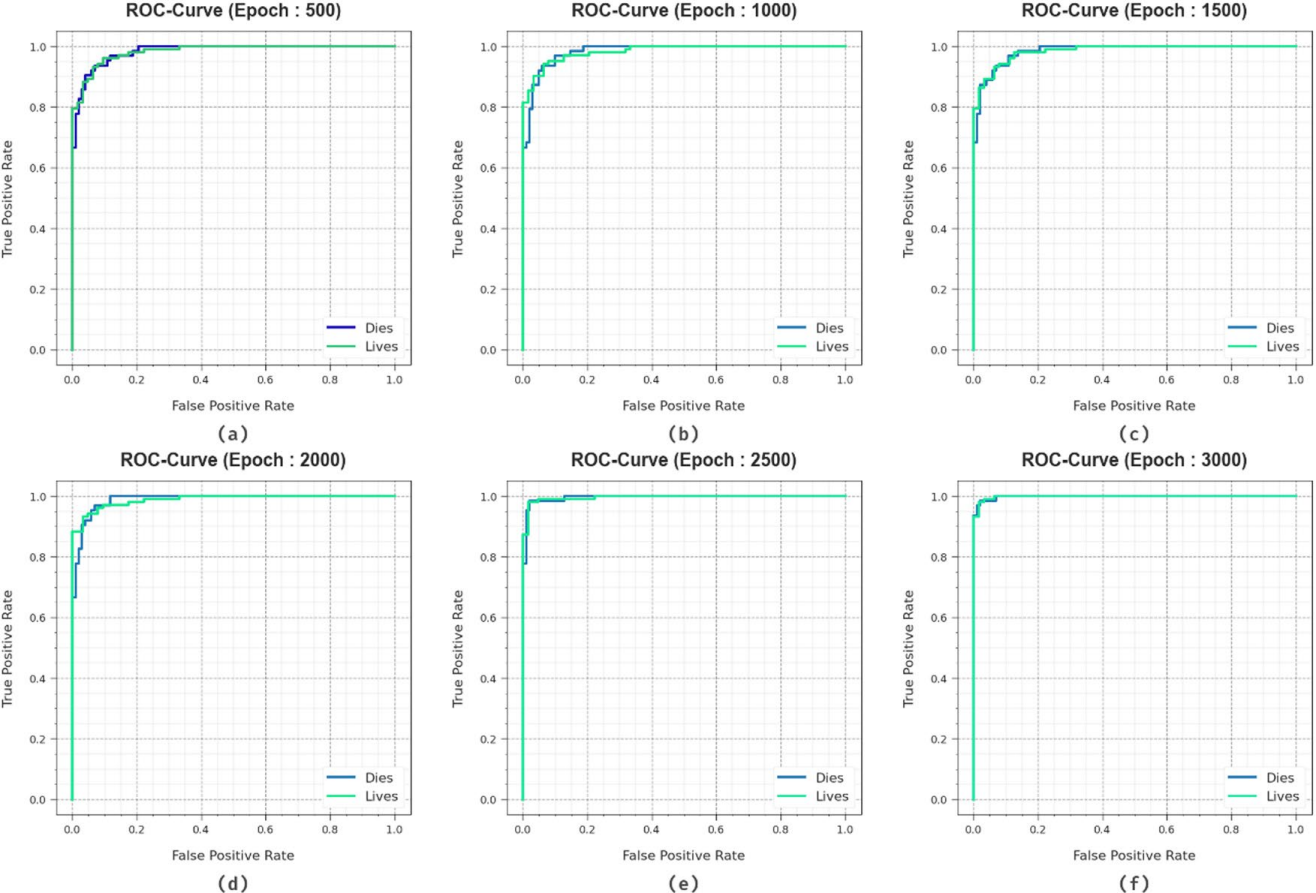
ROC curve of HCDAL-XAI method under (**a**-f) 500–3000 Epochs.

In Fig. 12, the ROC curve of the HCDAL-XAI method on various epochs is investigated. The solutions infer that the HCDAL-XAI method attains higher ROC outcomes in all class labels, demonstrating considerable proficiency in distinguishing the classes. This consistent pattern of increased values of ROC in numerous class labels denotes the efficacious results of the HCDAL-XAI approach in class prediction, underlining the classification process’s strong nature.

**Fig. 13:**
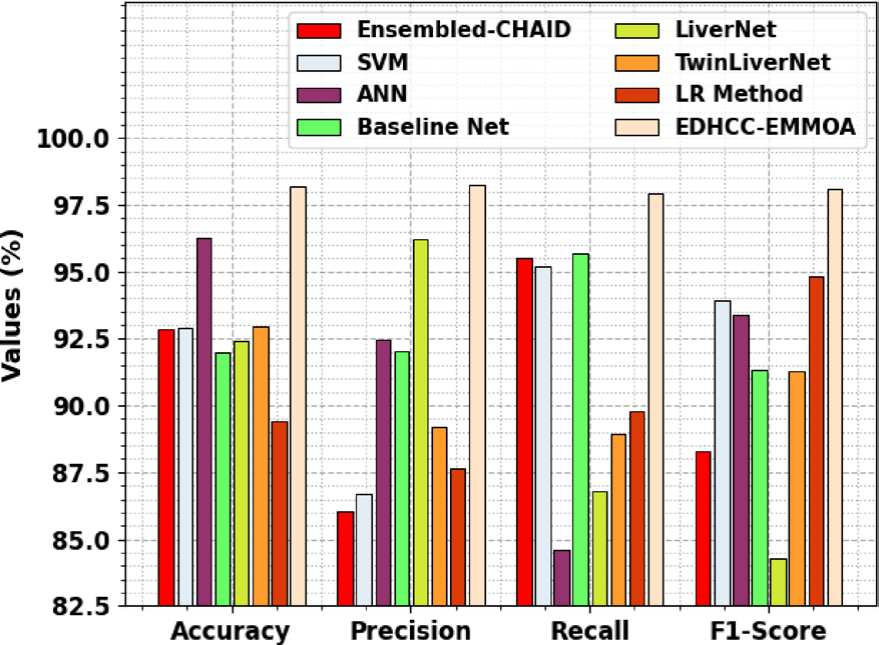
Comparative analysis of HCDAL-XAI approach with existing models.

The comparative analysis of the HCDAL-XAI approach with the current models is proven in Table 4; Fig. 13^36,37^. Based on $\:acc{u}_{y}$, the HCDAL-XAI approach has obtained a higher $\:acc{u}_{y}$ of 98.18%. At the same time, the Ensembled-CHAID, SVM, ANN, Baseline Net, LiverNet, TwinLiverNet, and LR methodologies have achieved a lesser $\:acc{u}_{y}$ of 92.84%, 92.91%, 96.25%, 91.99%, 92.44%, 92.94%, and 89.44%, respectively.

**Table 4:** Comparative analysis of the HCDAL-XAI approach with existing models.

Models	$\:\boldsymbol{A}\boldsymbol{c}\boldsymbol{c}{\boldsymbol{u}}_{\boldsymbol{y}}$	$\:\boldsymbol{P}\boldsymbol{r}\boldsymbol{e}{\boldsymbol{c}}_{\boldsymbol{n}}$	$\:\boldsymbol{R}\boldsymbol{e}\boldsymbol{c}{\boldsymbol{a}}_{\boldsymbol{l}}$	$\:{\boldsymbol{F}1}_{\boldsymbol{s}\boldsymbol{c}\boldsymbol{o}\boldsymbol{r}\boldsymbol{e}}$
Ensembled-CHAID	92.84	86.07	95.50	88.31
SVM	92.91	86.67	95.22	93.93
ANN	96.25	92.49	84.58	93.38
Baseline Net	91.99	92.04	95.69	91.34
LiverNet	92.44	96.19	86.80	84.30
TwinLiverNet	92.94	89.20	88.91	91.29
LR Method	89.44	87.66	89.80	94.80
EDHCC-EMMOA	98.18	98.22	97.92	98.07

Likewise, depending on $\:pre{c}_{n}$, the HCDAL-XAI approach has a higher $\:pre{c}_{n}$ of 98.22%. At the same time, the Ensembled-CHAID, SVM, ANN, Baseline Net, LiverNet, TwinLiverNet, and LR methodologies have obtained a lower $\:pre{c}_{n}$ of 86.07%, 86.67%, 92.49%, 92.04%, 96.19%, 89.20%, and 87.66%, correspondingly. Finally, under $\:{F1}_{score}$, the HCDAL-XAI approach has obtained a higher $\:{F}_{score}$ of 98.07%. At the same time, the Ensembled-CHAID, SVM, ANN, Baseline Net, LiverNet, TwinLiverNet, and LR methodologies have a lower $\:{F1}_{score}$ of 88.31%, 93.93%, 93.38%, 91.34%, 84.30%, 91.29%, and 94.80%. Thus, the proposed model is employed for accurate HCC detection and classification.

**Fig. 14:**
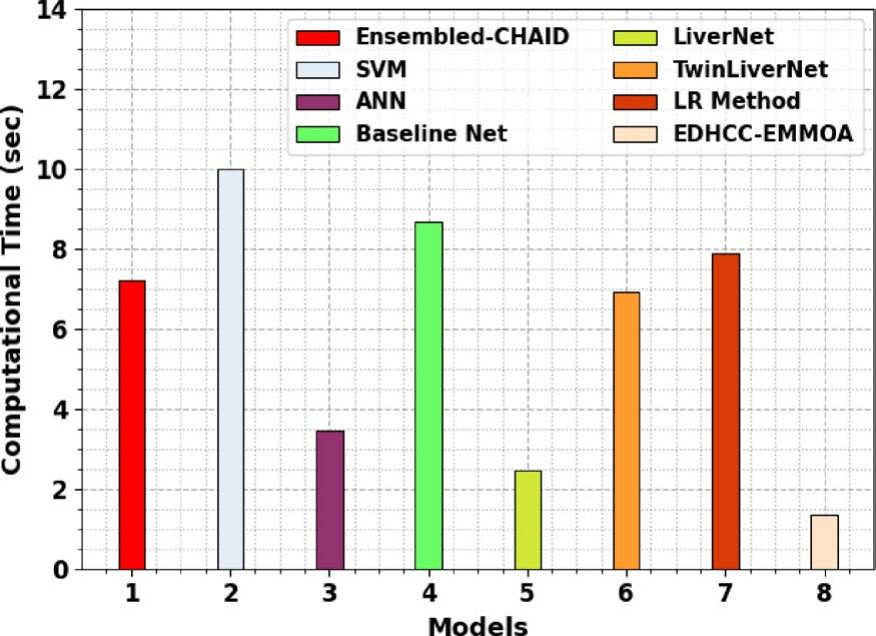
CT analysis of the HCDAL-XAI methodology with existing models.

Table 5; Fig. 14 depict the computational time (CT) analysis of the HCDAL-XAI methodology with existing models. The HCDAL-XAI methodology attained the fasest CT of 1.34 s, significantly lesser than other approaches such as Ensembled-CHAID at 7.20 s, Support Vector Machine (SVM) at 10.00 s, ANN at 3.46 s, Baseline Net at 8.69 s, LiverNet at 2.45 s, TwinLiverNet at 6.93 s, and LR method at 7.88 s. This demonstrates that the HCDAL-XAI model not only provides accurate predictions but also outperforms in rapid computation, making it highly appropriate for real-time liver disease diagnosis and practical clinical deployment.

**Table 5:** CT analysis of the HCDAL-XAI methodology with existing models.

Methodology	CT (sec)
Ensembled-CHAID	7.20
SVM	10.00
ANN	3.46
Baseline Net	8.69
LiverNet	2.45
TwinLiverNet	6.93
LR Method	7.88
EDHCC-EMMOA	1.34

**Fig. 15:**
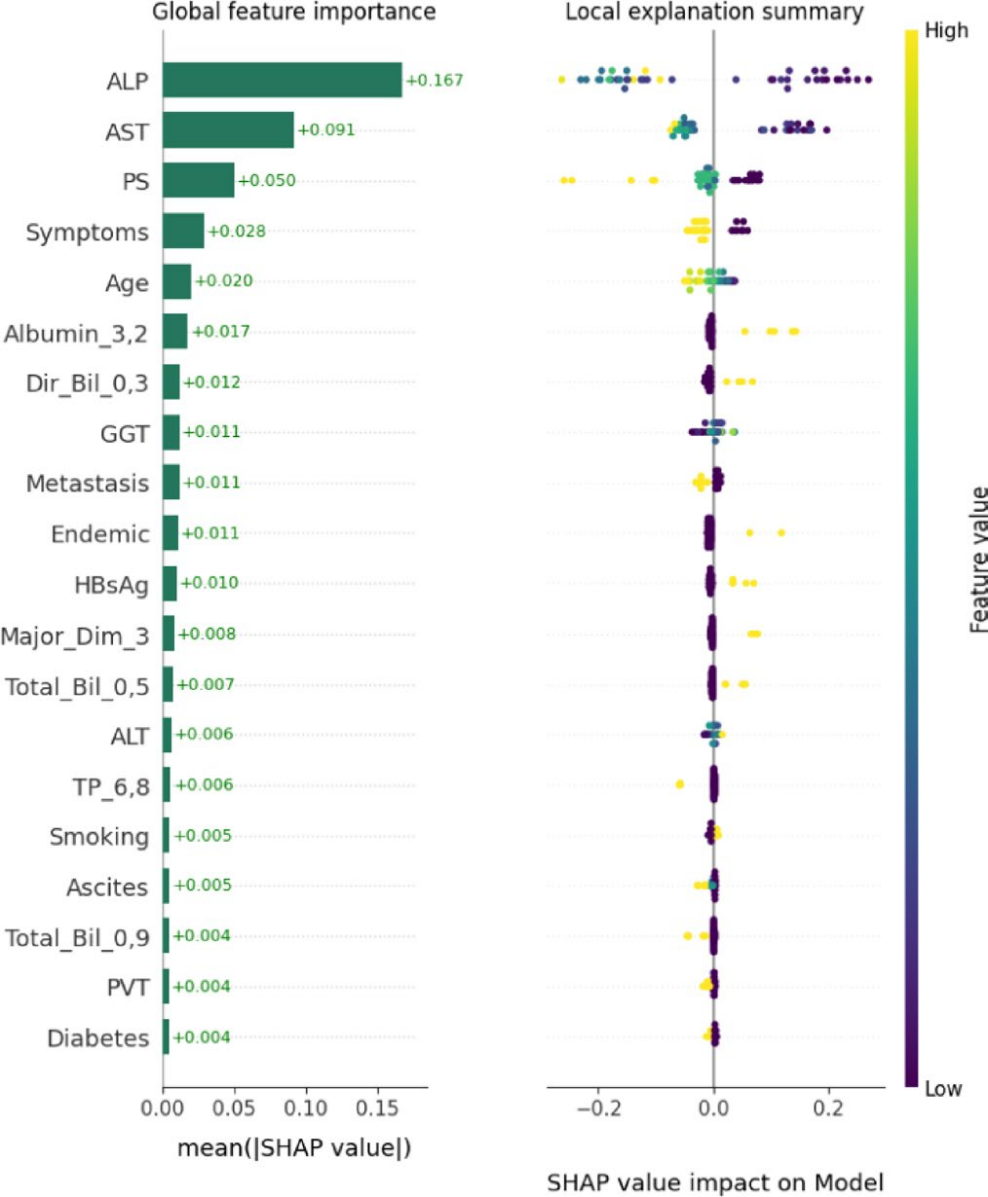
SHAP-based global and local interpretability of HCDAL-XAI model.

Figure 15 exhibits the SHAP-based global and local interpretability outcomes of the HCDAL-XAI system. The global feature significance plot displays that ALP and AST can have more implications features, providing the maximum mean absolute SHAP values such as Age, indications, and PS, representing their robust influence on the predictable model. Alternative medical and biochemical parameters namely Albumin, Direct Bilirubin, GGT, and Metastasis offer reasonably, while issues like Smoking, Ascites, PVT, and Diabetes are comparatively lesser impact. The local explanation summary exemplifies how individual feature values are affected predictions, where maximum feature values (yellow) or minimum values (purple) either upsurge or decline the model output.

Table 6 depicts the ablation study analysis of the HCDAL-XAI methodology. The SAE without GRU and DBN achieves an $\:acc{u}_{y}$ of 93.42%, $\:pre{c}_{n}$ of 92.89%, $\:rec{a}_{l}$ of 93.00%, and $\:{F1}_{score}$ of 93.37%. Furthermore, the GRU model without SAE and DBN improves performance with an $\:acc{u}_{y}$ of 94.94%, $\:pre{c}_{n}$ of 94.34%, $\:rec{a}_{l}$ of 94.39%, and $\:{F1}_{score}$ of 94.72%. Further enhancement is observed with the DBN model without GRU and SAE, which attains an $\:acc{u}_{y}$ of 96.16%, $\:pre{c}_{n}$ of 95.89%, $\:rec{a}_{l}$ of 95.89%, and $\:{F1}_{score}$ of 96.10%. The HCDAL-XAI model highlights the optimum performance, achieving an $\:acc{u}_{y}$ of 98.18%, $\:pre{c}_{n}$ of 98.22%, $\:rec{a}_{l}$ of 97.92%, and $\:{F1}_{score}$ of 98.07%, demonstrating the efficiency of integrating diverse DL models.

**Table 6:** Ablation study analysis of the HCDAL-XAI methodology.

Models	$\:\boldsymbol{A}\boldsymbol{c}\boldsymbol{c}{\boldsymbol{u}}_{\boldsymbol{y}}$	$\:\boldsymbol{P}\boldsymbol{r}\boldsymbol{e}{\boldsymbol{c}}_{\boldsymbol{n}}$	$\:\boldsymbol{R}\boldsymbol{e}\boldsymbol{c}{\boldsymbol{a}}_{\boldsymbol{l}}$	$\:{\boldsymbol{F}1}_{\boldsymbol{s}\boldsymbol{c}\boldsymbol{o}\boldsymbol{r}\boldsymbol{e}}$
SAE (Without GRU and DBN)	93.42	92.89	93.00	93.37
GRU (Without SAE and DBN)	94.94	94.34	94.39	94.72
DBN (Without GRU and SAE)	96.16	95.89	95.89	96.10
EDHCC-EMMOA (Ensemble DL models)	98.18	98.22	97.92	98.07

Table 7 indicates the efficiency analysis of the HCDAL-XAI model in terms of Floating Point Operations (FLOPs), Graphics Processing Unit (GPU), and inference time^38^. The HCDAL-XAI model demonstrated extremely lightweight and resource-efficient by illustrating only 0.05 G FLOPs, a GPU memory requirement of 952 MB, and an inference time of 0.0532 s. Moreover, the Unext and LinkNet models exhibited lesser performance. Compared to heavy models like U-Net, DeeplabV3+, AG-Net, and D1CD2U-Net, HCDAL-XAI method achieves a dramatic reduction in computational cost while maintaining competitive performance, making it highly appropriate for deployment in real-time and resource-constrained environments.

**Table 7:** Computational efficiency assessment of the HCDAL-XAI model.

Models	FLOPs	GPU	Inference Time
U-Net	54.82 G	3950 M	0.0264 s
FCN	25.50 G	4054 M	0.0193 s
DeeplabV3+	6.61 G	3533 M	0.0229 s
PSPNet	2.51 G	4605 M	0.0211 s
Unext	0.10 G	5545 M	0.0159 s
LinkNet	3.04 G	2661 M	0.0176 s
AG-Net	16.57 G	4700 M	0.0335 s
D1CD2U-Net	114.28 G	3571 M	0.1021 s
EDHCC-EMMOA	0.05 G	952 M	0.0532 s

## Conclusion

This manuscript has presented an automated model for HCC detection, named the HCDAL-XAI model, from biomedical data. The aim is to deliver an accurate detection model for initial diagnosis and efficient treatment of HCC using progressive methods. At first, the data pre-processing step uses min-max normalisation to change the input data into a useful pattern. Furthermore, the HCDAL-XAI model utilises an ensemble of SAE models, the GRU method, and the DBN technique for the classification process. Finally, the XAI technique applies SHAP to improve the transparency of AI techniques. The experimental evaluation of the HCDAL-XAI model occurs using a benchmark dataset. The empirical results indicated the enhanced performance of the HCDAL-XAI method compared to recent methodologies. The limitations include the relatively small dataset size, which may also limit the statistical strength of the results and restrict broader applicability. The dataset may also introduce bias across diverse clinical settings. The diagnostic capability is affected as the study concentrates on ured clinical data and does not incorporate imaging or genomic information. The study does not properly explore external validation on independent datasets, and in addition, real-time clinical deployment aspects are not addressed. Future work should concentrate on collecting larger, multi-centre datasets, integrating multimodal data, and performing extensive external validation. Further efforts will also explore clinical integration and prospective testing to improve real-world applicability.

## Data Availability

The data that support the findings of this study are openly available in [https://www.kaggle.com/datasets/mrsantos/hcc-dataset](https:/www.kaggle.com/datasets/mrsantos/hcc-dataset), reference number^35^.

## Code availability

The codes are freely available at GitHub (https://github.com/zgenz1537-code/HepatocellularCarcinomaDet.git).

## References

[1] Zhang, Z. M. et al. Early diagnosis of hepatocellular carcinoma using machine learning method. *Front. Bioeng. Biotechnol.* **8**, 254 (2020). 32292778 10.3389/fbioe.2020.00254 PMC7122481

[2] Ioannou, G. N. et al. Assessment of a deep learning model to predict hepatocellular carcinoma in patients with hepatitis C cirrhosis. *JAMA Netw. open.* **3** (9), e2015626–e2015626 (2020). 32870314 10.1001/jamanetworkopen.2020.15626 PMC7489819

[3] Ahn, J. C., Qureshi, T. A., Singal, A. G., Li, D. & Yang, J. D. Deep learning in hepatocellular carcinoma: current status and future perspectives. *World J. Hepatol.* **13**(12), 2039 (2021). 35070007 10.4254/wjh.v13.i12.2039 PMC8727204

[4] Saillard, C. et al. Predicting survival after hepatocellular carcinoma resection using deep learning on histological slides. *Hepatology* **72** (6), 2000–2013 (2020). 32108950 10.1002/hep.31207

[5] Sato, M. et al. Machine-learning approach for the development of a novel predictive model for the diagnosis of hepatocellular carcinoma. *Sci. Rep.* **9**(1), 7704 (2019). 31147560 10.1038/s41598-019-44022-8 PMC6543030

[6] Zhang, S. et al. Construction of a diagnostic model for hepatitis B-related hepatocellular carcinoma using machine learning and artificial neural networks and revealing the correlation by immunoassay. *Tumour Virus Res.* **16**, 200271 (2023). 37774952 10.1016/j.tvr.2023.200271 PMC10638043

[7] Shi, J. Y. et al. Exploring prognostic indicators in the pathological images of hepatocellular carcinoma based on deep learning. *Gut* **70** (5), 951–961 (2021). 32998878 10.1136/gutjnl-2020-320930

[8] Chung, D. Artificial intelligence in healthcare and medicine technology development review. *Eng. Appl. Artif. Intell.* **143**, 109801 (2025).

[9] Ala, A., Simic, V., Pamucar, D. & Bacanin, N. Enhancing patient information performance in internet of things-based smart healthcare system: Hybrid artificial intelligence and optimisation approaches. *Eng. Appl. Artif. Intel.* **131**, 107889 (2024).

[10] Hou, X. et al. Explainable deep learning model with the internet of medical devices for early lung abnormality detection. *Eng. Appl. Artif. Intel.* **153**, 110961 (2025).

[11] Rhyou, S. Y. & Yoo, J. C. Automated ultrasonography of hepatocellular carcinoma using discrete wavelet transform based deep-learning neural network. *Med. Image. Anal.* **101**, 103453 (2025). 39818008 10.1016/j.media.2025.103453

[12] Usha, S., Arulkarthick, V. J., Srihari, K. & Kumar, P. M. V. Hepatocellular carcinoma recognition from ultrasound images with pixelated disparity based deep CNN-based fire hawk optimiser. *Biomed. Signal Process. Control* **103**, 107401 (2025).

[13] Gu, Z. et al. Cell Segmentation With Globally Optimised Boundaries (CSGO): A Deep Learning Pipeline for Whole-Cell Segmentation in Hematoxylin-and-Eosin–Stained Tissues. *Lab. Investig.* **105**(2), 102184 (2025). 39528162 10.1016/j.labinv.2024.102184 PMC12305447

[14] Rajeev, D., Remya, S. & Nayyar, A. *HCCNet Fusion: a synergistic approach for accurate hepatocellular carcinoma staging using deep learning paradigm* 1–34 (Multimedia Tools and Applications, 2024).

[15] Lin, L., Liu, Y., Gao, M. & Rezaeipanah, A. Improving hepatocellular carcinoma diagnosis using an ensemble classification approach based on Harris Hawks Optimization. *Heliyon* **10**(1), e23497 (2024). 38169861 10.1016/j.heliyon.2023.e23497 PMC10758797

[16] Zhang, J. et al. *Hepatocellular carcinoma histopathological images grading with a novel attention-sharing hybrid network based on multi-feature fusion* Vol. 86, 105126 (Biomedical Signal Processing and Control, 2023).

[17] Feng, X. et al. *Diagnosis of hepatocellular carcinoma using a deep network with multi-view enhanced patterns mined in contrast-enhanced ultrasound data* Vol. 118, 105635 (Engineering Applications of Artificial Intelligence, 2023).

[18] Cinar, U., Atalay, C. & Cetin, Y. Y. Human hepatocellular carcinoma classification from H&E stained histopathology images with 3D convolutional neural networks and focal loss function. *J. Imag.* **9**(2), 25 (2023). 10.3390/jimaging9020025 PMC9959324 36826944

[19] Wang, Z. et al. Precision Strike Strategy for Liver Diseases Trilogy with Xiao-Chai-Hu Decoction: A Meta-Analysis with Machine Learning. *Phytomedicine* **142**, 156796 (2025). 40347886 10.1016/j.phymed.2025.156796

[20] Hu, E. et al. A novel microbial and hepatic biotransformation-integrated network pharmacology strategy explores the therapeutic mechanisms of bioactive herbal products in neurological diseases: the effects of Astragaloside IV on intracerebral hemorrhage as an example. *Chinese Med.* **18**(1), 40 (2023). 10.1186/s13020-023-00745-5 PMC10108474 37069580

[21] Men, X. et al. Activatable fluorescent probes for imaging and diagnosis of hepatocellular carcinoma. *J. Innov. Optic. Health Sci.* **18**(3), 2530004 (2025).

[22] Li, J. et al. Outlier detection using iterative adaptive mini-minimum spanning tree generation with applications on medical data. *Front. Physiol.* **14**, 1233341 (2023). 37900945 10.3389/fphys.2023.1233341 PMC10613083

[23] Savaş, S. Explainable Artificial Intelligence for Diagnosis and Staging of Liver Cirrhosis Using Stacked Ensemble and Multi-Task Learning. *Diagnostics* **15**(9), 1177 (2025). 40361994 10.3390/diagnostics15091177 PMC12071678

[24] Zarlashat, Y., Tayyeba, A. & Hussain, S. Neutrophil-to-lymphocyte and platelet-to-lymphocyte ratios in hepatocellular carcinoma: From inflammation to clinical applications. *Cancer Plus* **6**(4), 5758 (2024).

[25] Tang, T. et al. Interpretable machine learning model for predicting post-hepatectomy liver failure in hepatocellular carcinoma. *Sci. Rep.* **15**(1), 15469 (2025). 40316613 10.1038/s41598-025-97878-4 PMC12048636

[26] Zhang, Z. M. et al. Development of machine learning-based predictors for early diagnosis of hepatocellular carcinoma. *Sci. Rep.* **14**(1), 5274 (2024). 38438393 10.1038/s41598-024-51265-7 PMC10912761

[27] Peng, C. et al. Opportunistic detection of hepatocellular carcinoma using Noncontrast CT and deep learning artificial intelligence. *J. Am. Coll. Radiol.* **22** (3), 249–259 (2025). 40044303 10.1016/j.jacr.2024.12.011

[28] Li, J. et al. Interpretable machine learning based on CT-derived extracellular volume fraction to predict pathological grading of hepatocellular carcinoma. *Abdom. Radiol.* **49** (10), 3383–3396 (2024). 10.1007/s00261-024-04313-9 38703190

[29] Adnan, M. M. & Abhilash, P. K. Enhanced Convolutional Neural Network for Accurate Crop Recommendation System on Climate Data. In *SHS Web of Conferences* (Vol. 216, p. 01041). EDP Sciences. (2025).

[30] Siri, D. et al. Bio-inspired feature selection and graph learning for sepsis risk stratification. *Sci. Rep.* **15** (1), 1–17 (2025). 40404796 10.1038/s41598-025-02889-w PMC12098832

[31] Lin, X., Yin, S. & Li, X. A Trustworthy Artificial Intelligence Framework for Predicting Gasoline Octane Loss Using Sparse Autoencoder and Stacking Ensemble Learning in Petrochemical Processes. (2025).

[32] Wang, G., Xu, S., Chen, Z. & Li, Y. A Hybrid Model Integrating Variational Mode Decomposition and Intelligent Optimization for Vegetable Price Prediction. *Agriculture* **15**(9), 919 (2025).

[33] Munshi, R. & Gupta, S. K. G. Innovative diagnostic strategies for identifying High-Risk patients using laboratory parameters. *SGS-Engineering & Sciences*, **1**(1). (2025).

[34] Hanintya, D. L., Sukarno, P. & Wardana, A. A. Comparing explainable AI framework: study case on detection of DNS exfiltration attach using neural network. *Procedia Comput. Sci.* **269**, 1022–1032 (2025).

[35] https://www.kaggle.com/datasets/mrsantos/hcc-dataset

[36] Azit, N. A. et al. Prediction of hepatocellular carcinoma risk in patients with type-2 diabetes using supervised machine learning classification model. *Heliyon* **8**(10), e10772 (2022). 36203910 10.1016/j.heliyon.2022.e10772 PMC9529545

[37] Pino, C. et al. November. Twinlivernet: predicting TACE treatment outcome from CT scans for hepatocellular carcinoma using deep capsule networks. In *2021 43rd Annual International Conference of the IEEE Engineering in Medicine & Biology Society (EMBC)* (pp. 3039–3043). IEEE. (2021). 10.1109/EMBC46164.2021.9630913 34891884

[38] Zhang, C. et al. Liver Tumor Segmentation Based on Multi-Scale Deformable Feature Fusion and Global Context Awareness. *Biomimetics* **10**(9), 576 (2025). 41002810 10.3390/biomimetics10090576 PMC12467957

